# Predictive Factors for Chemoradiation-Induced Oral Mucositis and Dysphagia in Head and Neck Cancer: A Scoping Review

**DOI:** 10.3390/cancers15235705

**Published:** 2023-12-04

**Authors:** Alexander J. Nicol, Jerry C. F. Ching, Victor C. W. Tam, Kelvin C. K. Liu, Vincent W. S. Leung, Jing Cai, Shara W. Y. Lee

**Affiliations:** 1 Department of Health Technology and Informatics, The Hong Kong Polytechnic University, Hong Kong, China; alexander.nicol@connect.polyu.hk (A.J.N.); jerrycf.ching@connect.polyu.hk (J.C.F.C.); victorcw.tam@connect.polyu.hk (V.C.W.T.); chun-kit-kelvin.liu@connect.polyu.hk (K.C.K.L.); wsv.leung@polyu.edu.hk (V.W.S.L.); 2 The Hong Kong Polytechnic University Shenzhen Research Institute, Shenzhen 518000, China

**Keywords:** oral mucositis, dysphagia, head and neck cancer, predictive factors, acute toxicity, late toxicity

## Abstract

**Simple Summary:**

Head and neck cancer is the seventh-most prevalent cancer worldwide. Despite advances in treatment, many patients suffer from chemoradiation-induced oral mucositis and dysphagia, affecting both treatment outcome and quality of life. Accurate prediction of the severity of toxicities is important for optimizing management and improving patient outcomes. The aim of this scoping review was to provide recommendations for the improvement in predictive models for oral mucositis and dysphagia. This was achieved by comprehensively mapping the landscape of reported predictors and critically evaluating the performance, methodology, and reporting of predictive models for these conditions. Implementation of these improvements is desirable to enable the early detection of patients at high risk of severe toxicity, thereby offering opportunities for preemptive care, intensified support, and, ultimately, improved patient outcomes.

**Abstract:**

Despite advances in head and neck cancer treatment, virtually all patients experience chemoradiation-induced toxicities. Oral mucositis (OM) and dysphagia are among the most prevalent and have a systemic impact on patients, hampering treatment outcome and harming quality of life. Accurate prediction of severe cases is crucial for improving management strategies and, ultimately, patient outcomes. This scoping review comprehensively maps the reported predictors and critically evaluates the performance, methodology, and reporting of predictive models for these conditions. A total of 174 studies were identified from database searches, with 73 reporting OM predictors, 97 reporting dysphagia predictors, and 4 reporting both OM and dysphagia predictors. These predictors included patient demographics, tumor classification, chemoradiotherapy regimen, radiation dose to organs-at-risk, genetic factors, and results of clinical laboratory tests. Notably, many studies only conducted univariate analysis or focused exclusively on certain predictor types. Among the included studies, numerous predictive models were reported: eight for acute OM, five for acute dysphagia, and nine for late dysphagia. The area under the receiver operating characteristic curve (AUC) ranged between 0.65 and 0.81, 0.60 and 0.82, and 0.70 and 0.85 for acute oral mucositis, acute dysphagia, and late dysphagia predictive models, respectively. Several areas for improvement were identified, including the need for external validation with sufficiently large sample sizes, further standardization of predictor and outcome definitions, and more comprehensive reporting to facilitate reproducibility.

## 1. Introduction

In 2020, head and neck cancer (HNC) accounted for over 900,000 new cases globally, making it the seventh-most prevalent type of cancer [1]. It encompasses malignancies that originate in the oral cavity, lips, tongue, pharynx, larynx, salivary glands, as well as in the nasal cavity and sinuses, with some studies also including esophageal malignancies [2]. Primary treatments for HNC include surgery and radiotherapy (RT), supplemented by chemotherapy. Radiotherapy aims to halt tumor growth by inducing DNA damage in tumor cells using high-energy X-rays [3]. However, normal cells will also be destroyed in a similar manner, resulting in radiation-induced toxicity. The degree of tissue damage depends on total radiation dose, fractionation, and tissue properties [4]. Chemotherapy amplifies the overall toxicity through its effect on oral microbiota [5]. Most patients receiving chemoradiation in the head and neck region will experience moderate-to-severe toxicity.

The Radiation Therapy Oncology Group (RTOG) and National Cancer Institute Common Toxicity Criteria (NCI-CTC) define acute toxicity as occurring within 90 days of RT commencement [6]. Damage to normal tissue triggers inflammatory responses in the acute phase, followed by tissue repair and remodeling after treatment completion. Acute toxicities typically include OM, dysphagia, xerostomia, dysgeusia, odynophagia, and dermatitis. Most acute toxicities will resolve within weeks after treatment. Late toxicity, which may surface months or years later, results from irreversible damage to tissues and structures. Examples of late toxicities include dysphagia, xerostomia, osteoradionecrosis, myositis, dental caries, oral cavity necrosis, fibrosis, impaired wound healing, and lymphedema [7]. These are typically chronic conditions which require lifelong management. Two of the most prevalent toxicities are oral mucositis (OM) and dysphagia.

Oral mucositis (OM) is inflammation of the oral mucosa involving pain, soreness, and redness, which may develop into ulceration, hemorrhage, and necrosis. Severe OM requires narcotic-grade painkillers as part of the management strategy. OM may be graded according to its visual presentation, or by the severity of pain experienced by the patient [6,8,9]. OM is often accompanied by a fungal infection, oral candidiasis, which can further exacerbate pain and inflammation. The intensity of OM can be so severe that patients opt for treatment delays or discontinuation, significantly worsening their treatment outcome. Fesinmeyer et al. found that HNC patients who experienced an interruption in RT had an increased risk of death [10]. Prevention and treatment of OM are actively being investigated. Proposed interventions include dental care for improved oral hygiene, mouth rinses, topical agents, laser therapy, and growth factors [11].

Dysphagia refers to difficulty swallowing, which may be diagnosed according to different criteria. Assessment of the swallowing mechanism may be conducted using the modified barium swallow (MBS) or Videofluoroscopic Swallow Study (VFSS) or using Fiberoptic Endoscopic Evaluation of Swallowing (FEES). Alternatively, the grading of dysphagia can be evaluated based on dietary intake, weight loss thresholds, and indications for tube feeding [6,12]. Other complications from dysphagia include obstruction, perforation, and aspiration (inhalation of food into the airway or lungs). Treatments for dysphagia include dietary support, speech and language therapy for re-training swallowing, and surgery or medication for alleviation of the narrowing and stiffness of the esophagus. To avoid consequent weight loss, nutritional support can be provided through liquid food supplements or tube feeding. In very severe cases, more aggressive interventions such as percutaneous gastrostomy or parenteral feeding may be used.

OM and dysphagia, while common, pose severe threats to patients’ well-being. In a study on 212 HNC patients undergoing IMRT, a staggering 50% suffered from moderate-to-severe OM, while a concerning 75% faced moderate-to-severe dysphagia [13]. Alarmingly, in a study focusing on older HNC patients, 9% were hospitalized or sought emergency care due to acute OM toxicity, and an even higher 19% for dysphagia-related issues [14]. Patients suffering from severe toxicity may be unable to attend their regular treatment fractions because of pain. In addition, patients suffer severely reduced QoL. The pain and discomfort which directly result from these toxicities are combined with the frustration over impairments to everyday actions such as eating and drinking. Interventions to maintain nutrition, such as nasogastric tube feeding, can also induce discomfort. The impact on QoL should not be underestimated, with these conditions inflicting a toll on the patient’s physical, emotional, and psychosocial health.

Accurate prediction of severe treatment-related toxicity among HNC patients is beneficial, allowing individualized management and enhancing patient outcomes. This scoping review aimed to systematically map the current literature on predictive factors for chemoradiation-induced OM and dysphagia among HNC patients, providing a quantitative as well as qualitative synthesis. This study sought to address two primary research questions: (1) Which factors are recognized as predictors for treatment-induced OM and dysphagia in HNC patients? (2) How efficacious are the prevailing predictive models in forecasting the severity of these toxicities? This review synthesizes current evidence to offer clinicians and researchers insights for enhancing predictive model development.

## 2. Materials and Methods

**Figure 1:**
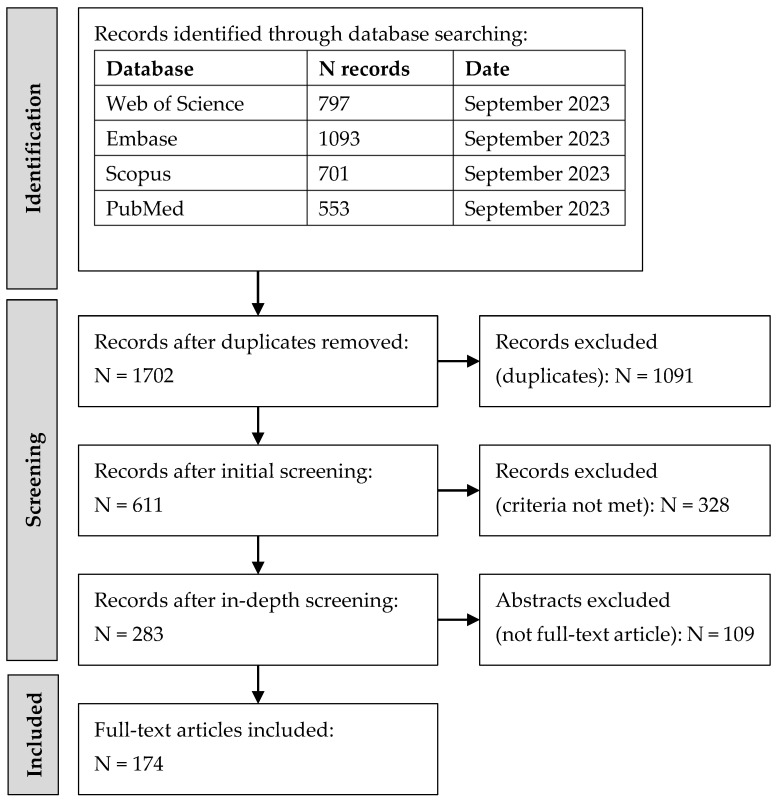
Flow diagram of selection of sources of evidence.

To identify studies on predictive factors for chemoradiation-induced OM and dysphagia in HNC patients, a systematic literature search was conducted in accordance with the Preferred Reporting Items for Systematic Review and Meta-Analysis Protocols Extension for Scoping Reviews (PRISMA-ScR) guidelines [15]. Data were collected through Embase, PubMed, Scopus, and Web of Science from year 2000 to September 2023. The detailed search strategy for each database is provided in Table A1. A flowchart of the study search and screening process is illustrated in Figure 1.

Eligible studies included those that reported predictive factors for OM or dysphagia. If both toxicities were reported, separate records were created for each. The outcome measures related to the severity, incidence, or duration of OM or dysphagia. Measures of dysphagia included objective and subjective severity scales and indicators including diagnoses of stricture or aspiration based on videofluoroscopy or modified barium swallow. Aspiration pneumonia, as a secondary consequence of dysphagia, was not included as an outcome measure. Included studies reported statistically significant factors or predictive models for the outcome. Study subjects were restricted to patients with head and neck cancer who received radiotherapy and/or chemotherapy. Consequently, studies using animal or in vitro models were excluded. Studies with 10 or fewer subjects were excluded, as were those not available in English, or those published before the year 2000.

After removing duplicates, screening was performed in two phases. The first phase involved screening by the title and abstract, followed by full-text screening in the second phase. Any non-full-length articles were excluded, due to limited detail and lower quality of evidence. A critical appraisal of individual sources of evidence was not conducted, considering the diversity in study designs and the volume of the literature included. Moreover, the primary purpose of the review was to map the existing research rather than assess the level of evidence of each study.

Data charting was conducted separately for OM and dysphagia. For each outcome, details of each study were tabulated in Tables S1 and S2. Data items included sample size, treatment regimen, outcome measure, outcome incidence, and timeframe. Summary statistics and overall incidences are reported in Table 1 and Table 2, respectively. Factors reported to be significantly correlated with the outcome (*p*-value < 0.05) were recorded under the appropriate category, along with an indication of whether the factor was significant in univariate analysis, multivariate analysis, or was reported as a model feature. To quantify the amount of evidence for each factor, the number of studies reporting it as significant in multivariate or model analysis was calculated for each toxicity outcome. Univariate analyses were not included, because of concerns over multiple testing biases and confounding variables. Factors for each toxicity outcome were grouped by factor type and ranked by the number of studies reporting it as significant in multivariate or model analysis. The number of studies reporting each factor as significant in any analysis (univariate, multivariate, or model) was also included for comparison.

Additionally, a subset of the included studies that reported predictive models for OM or dysphagia were investigated. Studies were included in this subset if they provided some form of validation performance score. The time frame, endpoint definition, model features, sample size, and validation type were recorded, along with the test performance score. This was typically reported in the form of the area under the receiver operating characteristic curve (AUC). An AUC close to 0.5 indicated that the prediction was equivalent to random chance, while a value close to 1.0 indicated a perfect prediction.

## 3. Results

### 3.1. Identification and Selection of Studies

The literature search resulted in 1702 unique records, of which 1528 were excluded during the screening process (Figure 1). A total of 174 full-text articles were included for this review. Seventy-three articles reported predictors for OM [16,17,18,19,20,21,22,23,24,25,26,27,28,29,30,31,32,33,34,35,36,37,38,39,40,41,42,43,44,45,46,47,48,49,50,51,52,53,54,55,56,57,58,59,60,61,62,63,64,65,66,67,68,69,70,71,72,73,74,75,76,77,78,79,80,81,82,83,84,85,86,87,88], four reported predictors for both OM and dysphagia [89,90,91,92], and ninety-seven reported predictors for dysphagia alone [93,94,95,96,97,98,99,100,101,102,103,104,105,106,107,108,109,110,111,112,113,114,115,116,117,118,119,120,121,122,123,124,125,126,127,128,129,130,131,132,133,134,135,136,137,138,139,140,141,142,143,144,145,146,147,148,149,150,151,152,153,154,155,156,157,158,159,160,161,162,163,164,165,166,167,168,169,170,171,172,173,174,175,176,177,178,179,180,181,182,183,184,185,186,187,188,189].

### 3.2. Overview of Included Studies

Table 1 summarizes the 174 full-text articles included in this review. Some articles reported predictors for both OM and dysphagia, so were treated as separate records in each analysis. The median number of HNC patients in each study was similar for OM (*n* = 91) and dysphagia (*n* = 100). The overall frequency of radiotherapy, chemotherapy, and surgery as treatments is also listed. Almost all the patients received radiotherapy, and most patients received chemotherapy. It should be noted that 81% of studies did not report the incidence of surgery as a treatment, so the proportion of patients with surgical history for OM studies (54%) and dysphagia studies (24%) may be inaccurate. Clinician-rated outcomes were most reported for both OM and dysphagia. OM was almost exclusively investigated in the acute period using clinician-rated gradings. Dysphagia was investigated at both the acute and late periods, with the majority focused on late dysphagia. Note that some studies reported predictors for both acute and late toxicities, and some without the timeframe specified. Among studies on OM, 59% included only univariate analysis, while 41% utilized multivariate analysis or developed a predictive model. Among studies on dysphagia, 37% included only univariate analysis, while 63% utilized multivariate analysis or developed a predictive model.

Table 2 describes the incidence of the top-reported OM and dysphagia outcomes. The most frequently reported OM outcome was RTOG/CTCAE/WHO grade 3+ (severe). Approximately 56% and 42% of patients endured grade 2+ (moderate-or-higher) and grade 3+ (severe-or-higher) OM during their treatment, respectively. Among dysphagia outcomes, severe dysphagia (as indicated by tube feeding or RTOG/CTCAE grade 3+) was very common in the acute period.

Table 3, Table 4 and Table 5 detail the analysis of predictive factors for acute OM, acute dysphagia, and late dysphagia, respectively. Univariate analysis does not account for relationships between factors and may be influenced by confounding. Therefore, factors were ranked by the number of studies that identified them as significant in multivariate analysis or predictive models. The factors can be classified into seven categories: patient, tumor, treatment, organ-at-risk dose, clinical laboratory test results, genetic expression, and “other”. Patient factors include demographics; tumor factors include TNM staging and tumor site; and treatment factors include treatment modalities and regimen. Dose factors pertain to the radiation delivered to the organ-at-risk; genetic factors refer to single-nucleotide polymorphisms of genes which were found to be correlated with toxicity; and clinical laboratory test results include the results of blood, saliva, or stool tests.

### 3.3. Predictors of Oral Mucositis

For acute oral mucositis (OM, Table 3), smoking was the patient factor most frequently reported as significant in multivariate analysis, followed by sex, body mass index, and age. Other factors included weight loss, performance status score, and number of teeth. Alcohol was reported by two studies in univariate analysis. The tumor factors most reported in multivariate analysis were tumor site, T-stage, and N-stage. Interestingly, the primary tumor volume was reported by one study in univariate analysis. Treatment factors were led by use of concurrent chemotherapy, followed by chemotherapy drug, RT fractionation, neoadjuvant chemotherapy, retropharyngeal lymph node irradiation, RT delivery time, RT field size, RT modality, and surgery-related factors. The dose factor most reported in multivariate analysis was the radiation dose to the oral cavity or extended oral cavity, followed by the dose to the oral mucosal surface, parotid glands, and pharyngeal space. Additionally, the dose to the tongue and the dose to the pharyngeal constrictor muscles were identified in univariate analysis. Many studies investigated the role of clinical laboratory test results, including white blood cell lymphocyte count, erythrocyte sediment rate (ESR), γ-H2AX (protein marker), and presence of the candida fungus. The roles of the RADIODTECT blood assay, epidermal growth factor, and neutrophil-to-lymphocyte ratio were also reported. Fourteen studies reported genetic factors as predictors of OM. However, only four adopted multivariate analysis. Tumor necrosis factor alpha was reported by two studies, but with different genotypes reported by each study (TT and GG) [33,70]. Single-nucleotide polymorphisms of XRCC1, a gene involved with DNA repair, were reported by two studies [51,81]. The remaining ten studies that reported genetic factors each returned a different factor. Beyond the previously mentioned categories, one study crafted a prediction model employing radiomics and dosiomics features derived from the primary tumor volume [23]. Two studies highlighted a significant correction between bioelectrical impedance measurements and OM in univariate analyses [34,72], while another study identified perfusion parameters as a significant determinant in a univariate analysis [69]. The most robust factors, significant in multivariate or model analysis, include RT dose to the oral cavity/oral mucosa, concurrent chemotherapy, smoking, tumor site, gender, and RT dose to the parotid glands.

### 3.4. Predictors of Dysphagia

Regarding acute dysphagia (Table 4), the most reported patient factor in multivariate analysis was age, followed by body mass index and performance status score. In terms of tumor factors, T-stage was the most reported, followed by tumor site and N-stage. For treatment factors, the most reported was the use of concurrent chemotherapy, followed by RT fractionation, chemotherapy drug, and neck irradiation regimen. The most reported dose factor was the accumulated radiation dose to the pharyngeal constrictor muscles, specifically the superior and inferior pharyngeal constrictors. This was followed by the dose to the medial constrictor, oral cavity or oral mucosa, parotid glands, larynx, and esophageal inlet or cricopharyngeus. Three studies [92,93,184] reported genetic factors regarding single-nucleotide polymorphisms. Two studies [89,170] reported clinical laboratory test results, including the presence of oral candidiasis and the result of the RADIODTECT blood assay. The most well-supported factors, significant in multivariate or model analysis, were T-stage, concurrent chemotherapy, tumor site, RT dose to constrictor muscles, N-stage, and patient age.

With respect to late dysphagia (Table 5), the most common patient factor in multivariate analysis was age, followed by smoking history, and baseline or acute weight loss. The most reported tumor factor was T-stage, followed by tumor site and N-stage. The most reported treatment factor was the use of concurrent chemotherapy, followed by surgery-related factors, RT fractionation, and neck irradiation regimen. The most reported dose factor was the radiation dose to the pharyngeal constrictor muscles, specifically the superior pharyngeal constrictor, dose to the larynx, medial and inferior constrictors, esophageal inlet or cricopharyngeus, oral cavity or oral mucosa, parotid glands, tongue or base of tongue, and esophagus. The doses received by the inferior brain stem and the submandibular glands were also reported. HPV status was the only clinical laboratory test result factor identified [159]. No genetic factors were identified as significantly correlated with late dysphagia. The most well-supported factors, significant in multivariate or model analysis, were RT dose to constrictor muscles, T-stage, patient age, tumor site, RT dose to larynx, concurrent chemotherapy, and N-stage.

### 3.5. Predictive Models for Oral Mucositis and Dysphagia

Table 6 and Table 7 present predictive models from the included studies that underwent either internal or external validation, providing insights into the current predictive performance of published models. Internally validated models were evaluated using hold-out test sets, cross-validation, or bootstrapping comprising samples from the same center as the training set. External validated models were evaluated on a hold-out test set taken from a separate center to assess the generalizability of the model. The details of the outcome measure, model features, and type of validation are also tabulated.

All the validated predictive models for OM were specific to the acute period. Severe-or-higher OM (≥grade 3), as scored by RTOG, CTCAE, or WHO, was used as an outcome by 6 out of 8 models [17,18,19,20,21,23]. Alternatively, an increase in RTOG grade from mild (grade 1) to moderate (grade 2) was used by Zhu et al. [16] and DAHANCA grade 3+ was used by Hansen et al. [22]. The validation performance, as measured by AUC, ranged from 0.65 to 0.81. Clinical features used in the models include sex, age, BMI, tumor site, N-stage, use of chemotherapy, chemotherapy drug, number of cycles of neoadjuvant chemotherapy, treatment acceleration, retropharyngeal lymph node irradiation, and treatment dose parameters. Dose volume histogram (DVH) parameters used in the models included dose to the oral cavity and dose to the mucosa surface contour. Zhu et al., reported a model using genetic information from oral bacteria [16]. Dong et al., reported a model using MR and CECT radiomic features extracted from the gross tumor volume [23]. The model size ranged between 2 and 19 features.

Among the validated predictive models for dysphagia, five predicted acute dysphagia and nine predicted late dysphagia. For acute dysphagia, outcomes were defined as tube feeding dependence [94,95,96] or CTCAE grading severe or higher (≥grade 3) [93,97]. The validation performance, as measured by the AUC, ranged from 0.60 to 0.82. Clinical features used in the models included sex, age, BMI, texture-modified diet, tumor site, T-stage, N-stage, performance status, pre-treatment weight loss, use of chemotherapy versus radiotherapy alone, use of concurrent chemotherapy, use of induction chemotherapy, and chemotherapy drug. DVH parameters used in the models included dose to the superior and inferior pharyngeal constrictor muscles, dose to the pharyngeal mucosa, dose to the contralateral parotid gland, dose to the oral cavity, and dose to the contralateral submandibular gland. De Ruyck et al. also incorporated a genetic polymorphism feature into their model [93]. The model size ranged from a single feature up to 20 features.

For late dysphagia, outcomes were defined as tube feeding dependence [103,106], occurrence of a dysphagia criterion (including tube feeding, aspiration, stricture, aspiration pneumonia) [99,104,105], RTOG/CTCAE moderate-or-higher dysphagia (≥grade 2) [100,101,102], or improvement in dysphagia grading [98]. The validation performance, as measured by the AUC, ranged from 0.70 to 0.85. Clinical features used in the models included age, T-stage, N-stage, tumor site, HPV status, smoking status, baseline weight loss, baseline dysphagia score, treatment acceleration, use of chemotherapy versus radiotherapy alone, neck dissection, and total dose to tumor. DVH parameters used in the models included dose to the pharyngeal constrictor muscles, dose to the larynx, dose to the contralateral parotid, dose to the cricopharyngeal muscle, dose to the mylogeniohyoid, and dose to the oral cavity. The model size ranged from a single feature up to nine features.

## 4. Discussion

To our knowledge, this is the first scoping review to map the current literature on predictors of and predictive models for the severity of OM and dysphagia in HNC patients. One hundred and seventy-four studies were included in this review. The reported predictors were categorized, grouped by toxicity and timeframe, and the numbers reported in univariate and multivariate analysis were analyzed (Table 3, Table 4 and Table 5). Additionally, eight, five, and nine studies that reported predictive models for the severity of acute OM, acute dysphagia, and late dysphagia were analyzed, respectively (Table 6 and Table 7).

### 4.1. Predictors of OM and Dysphagia

A broad range of predictors for the severity of OM and dysphagia have been identified, indicating the multifactorial and complex etiology of these conditions. Ranking predictors by the number of studies where they were significant in multivariate analysis is indicative of the quantity of evidence per predictor. Some predictor types, such as genetic factors for OM, were frequently reported as significant in univariate analyses, but not in multivariate analyses. For example, genome-wide association studies reported genetic variants associated with acute OM [74,86]. Even in other studies where multivariate analysis was performed, a limited set of predictor types were included. Further investigation is required to confirm the independent value of predictors and identify combined or interactive effects among a comprehensive range of predictor types.

### 4.2. Performance of Predictive Models

Predictive models mostly focused on severe toxicity, indicating their intended use for identifying high-risk patients who can be targeted for closer monitoring and more aggressive preventative measures. For acute OM, eight models were identified with AUCs ranging from 0.65 to 0.81 [16,17,18,19,20,21,22,23]. Five models emerged for acute dysphagia (AUC: 0.60–0.82) [93,94,95,96,97], and nine for late dysphagia (AUC: 0.70–0.85) [98,99,100,101,102,103,104,105,106]. A considerable variability in model performance was evident, suggesting opportunities for further improvement. The best performing models tended to incorporate multiple predictor types. For example, for OM, Hansen et al. included treatment acceleration alongside DVH parameters of the extended oral cavity [22], and Dong et al., included texture features from multiple imaging modalities [23]. For dysphagia, Dean et al., included patient, tumor, treatment, and DVH parameters of the pharyngeal mucosa [97]. Wopken et al., included tumor, treatment, and DVH parameters from multiple organs at risk [106]. Investigation of a broader range of factor types offers the potential to capture more of the multifactorial nature of these toxicities and further improve performance, provided that the challenges of increased dimensionality and interactive effects can be addressed.

### 4.3. Limitations of Predictive Models

External validation on data from a separate center provides a higher level of evidence. However, less than one third of the models were externally validated. Many of the studies reporting these models highlighted the lack of external validation and small sample size as limitations.

One of the main challenges involved in developing a predictive model for OM or dysphagia is in its generalizability to other clinical centers. The differences between centers may explain why only 27% of the studies utilized external validation. The grading system used for assessing toxicity can vary, as can the criteria for interventions such as tube feeding, a common outcome measure for dysphagia. For example, Dean et al. observed a difference in the scoring system for dysphagia between their training data and their validation data [97], and Willemsen et al., noted that individual and institutional preferences in feeding tube insertion policy may affect the apparent incidence of dysphagia [94]. Furthermore, the treatment regimen can also vary between centers. For example, there may be different guidelines for the use of neoadjuvant, concurrent, or adjuvant chemotherapy depending on tumor site and staging, and different guidelines for the choice of chemotherapy drug or radiation delivery. Sharabiani et al. recognized that the normal tissue complication probability (NTCP) models they used were not fully up to date with current treatment regimens, and so the generalizability of the models would be reduced [18]. The contouring of organs at risk (OARs) may also vary between centers, especially for organs which are not commonly delineated during standard practice such as the oral mucosa surface [19].

Models generally did not incorporate all types of predictors. For example, clinical laboratory test results and genetic factors were underrepresented in the models and may offer the potential to enhance prediction. For example, blood tests have an established role in patient monitoring. Information such as blood cell counts and the presence of inflammatory markers have been highlighted as potential predictors of severe toxicity. The incorporation of factors such as blood group type and its relationship with head and neck cancer subtypes may also offer the potential for more personalized models [190]. Regarding genetic factors, Hansen et al., suggest that characterizing normal tissue radiosensitivity through genomic or microbiomic data might improve the prediction of OM [22]. Regarding the role of DVH parameters, some studies did not include all relevant OARs in their analysis, such as the oral cavity for OM [23]. In some models, social factors such as smoking status and alcohol use were omitted [97]. Additionally, Dean et al. suggested that subjective patient-reported factors such as pain tolerance should also be investigated [97].

Another limitation was in terms of the reporting. Most studies did not display the receiver-operator curve for the validation set, preventing the comparison of sensitivity and specificity across different prediction thresholds. Where performance metrics were reported, it was sometimes unclear whether the value was applied to a training set or validation set. Moreover, insufficient information was often provided to independently validate the findings. Such information might include definitions of all model features, coefficient values, and model hyperparameters.

### 4.4. Recommendations for Future Model Development

Based on the limitations identified in the studies reporting predictive models, some recommendations are warranted. Studies should endeavor to recruit sufficiently large sample sizes to better identify patterns and reduce the impact of overfitting. Models should be externally validated to achieve a higher level of evidence, though the differences between centers should also be identified and discussed. Study methodology should be reported comprehensively, including details on patient selection criteria, variable and outcome definitions, preprocessing, feature selection, and the validation approach. Guidelines for OAR delineation should be followed wherever possible to facilitate reproducibility. Likewise, a greater standardization in the reporting of results is also desirable. Sufficient information should be provided to reproduce the model for validation purposes.

Certain types of predictors merit further investigation, particularly the role of clinical laboratory tests and genetic factors. Furthermore, the exploration of radiomic and dosiomic features may be beneficial through their ability to quantify textural properties and spatial dose distribution within OARs. It should be noted that toxicity has a subjective component. While most of the studies in this review have investigated clinician-rated toxicity, further exploration of the patient-reported toxicity outcomes and psychosocial factors as predictors of severe toxicity is warranted.

Future development of the predictive models for OM and dysphagia should include prospective studies. These may allow for a more comprehensive range of predictors to be measured and would improve the level of evidence by reducing the risk of selection bias. However, cross-institutional prospective studies would still face issues from differences in toxicity grading and treatment regimen between centers. This presents a bottleneck in the further development of models to predict severe toxicity.

### 4.5. Limitations of this Review

A limitation of this review is the broad definition of dysphagia, which includes not just dysfunction in the swallowing mechanism but also impaired oral intake and an indication for tube feeding, which in turn is often determined by weight loss. Analyzing predictors for each aspect separately might yield more specific findings. However, collecting a range of specific dysphagia outcomes is not typical in clinical practice, so the quantity of results would be reduced.

## 5. Conclusions

After reviewing 174 studies on OM and dysphagia, predictors were systematically assessed. Discrepancies observed between the findings from univariate and multivariate analyses suggest the need for deeper investigation into the relationships between different predictors. While several predictive models for the severity of OM or dysphagia have been proposed, the variability in their performance indicates potential for enhancement. This review identified several areas for improvement. Future studies should prioritize larger sample sizes, external validation, standardized predictor and outcome definitions, and comprehensive reporting to facilitate reproducibility. A broad range of predictor types should be collected to capture the multifactorial etiology of OM and dysphagia. The careful design of prospective studies will mitigate selection bias and allow some of the challenges of obtaining standardized and comprehensive predictor data to be overcome.

## Figures and Tables

**Table 1:** Summary statistics of included studies.

Summary Statistic	OM	Dysphagia
Full-text articles (N)	77	101
Patient cohort size (median)	91	100
Patient cohort with radiotherapy history (%)	100	100
Patient cohort with chemotherapy history (%)	80	88
Patient cohort with surgical history (%)	54	24
Clinician-rated outcome (%)	85	84
Patient-reported outcome * (%)	4	21
Investigated acute toxicity (%)	80	40
Investigated late toxicity (%)	1	62
Studies with univariate analysis only (%)	59	37

* Patient-reported outcome refers to toxicity outcomes defined by results of questionnaires or scales completed by the patient, such as pain rating, oral intake, swallowing ability, or quality-of-life scales.

**Table 2:** Toxicity outcome incidences.

Toxicity	Timeframe	OM Outcome	Incidence	Reported by N Studies
OM	Acute	RTOG/CTCAE/WHO grade 3+	42%	47
		RTOG/CTCAE/WHO grade 2+	56%	8
Dysphagia	Acute	Tube feeding(use/indication/dependence)	37%	13
		RTOG/CTCAE grade 3+	37%	11
		RTOG/CTCAE grade 2+	49%	2
	Late	Tube feeding(use/indication/dependence)	17%	14
		RTOG/CTCAE grade 3+	23%	7
		RTOG/CTCAE grade 2+	28%	4

**Table 3:** Number of studies demonstrating significant correlations between acute OM and various factors.

Factor Type	Factor	Multivariate or Model	All Analyses
Clinical laboratory tests	Blood, saliva, or stool properties	13	25
Dose	RT dose to oral cavity (entire volume)	9	10
	RT dose to oral mucosa (surface only)	6	7
	RT dose to parotid glands	3	3
	RT dose to pharyngeal space	1	1
	RT dose to constrictor muscle	0	1
	RT dose to tongue	0	1
Treatment	Concurrent chemotherapy	8	13
	Chemotherapy drug	2	2
	RT fractionation	2	3
	Neoadjuvant chemotherapy	2	2
	Retropharyngeal lymph node irradiation	1	1
	RT delivery time	1	3
	RT field size	1	1
	RT modality	1	1
	Surgery-related factors	1	2
	Number of chemotherapy cycles	0	1
	Use of tongue immobilizer	0	1
Patient	Smoking	7	10
	Sex	4	6
	Body mass index	3	6
	Age	3	9
	Baseline weight loss	2	3
	Performance status score	1	3
	Number of teeth	1	1
	Alcohol-related	0	2
Tumor	Tumor site	5	7
	T-stage	3	5
	N-stage	3	3
	Primary tumor volume	0	1
Genetic	Genetic factors	4	14
Other	Radiomics/dosiomics features	1	1
	Bioelectrical impedance measurement	0	2
	Perfusion/blood flow measurement	0	1

**Table 4:** Number of studies demonstrating significant correlations between acute dysphagia and various factors.

Factor Type	Factor	Multivariate or Model	All Analyses
Tumor	T-stage	9	11
	Tumor site	8	14
	N-stage	6	8
Treatment	Concurrent chemotherapy	9	11
	RT fractionation	4	7
	Chemotherapy drug type	3	4
	Neck irradiation regimen	3	6
	RT field size	2	3
	Surgery-related factors	2	2
	Adjuvant chemotherapy	1	1
	Brachytherapy	1	1
	Neoadjuvant chemotherapy	1	1
	RT modality	1	2
Dose	RT dose to constrictor muscles	7	12
	RT dose to inferior pharyngeal constrictor (IPC)	5	6
	RT dose to superior pharyngeal constrictor (SPC)	5	8
	RT dose to middle pharyngeal constrictor (MPC)	3	5
	RT dose to oral cavity volume/oral mucosa surface	3	4
	RT dose to parotids	3	3
	RT dose to larynx	2	5
	RT dose to esophageal inlet/cricopharnygeus	2	3
	RT dose to esophagus	1	2
	RT dose to pharyngeal mucosa	1	1
	RT dose to pharynx	1	1
	RT dose to submandibular glands	1	1
	RT dose to primary tumor	1	1
Patient	Age	6	8
	Body mass index	4	4
	Performance status score	4	5
	Baseline weight loss	3	5
	Sex	3	5
	Smoking history	3	5
	Pretreatment dysphagia	2	4
	Constrictor muscle geometry	1	1
Clinical laboratory tests	Blood or saliva properties	2	2
Genetic	Genetic factors	3	3

**Table 5:** Number of studies demonstrating significant correlations between late dysphagia and various factors.

Factor Type	Factor	Multivariate or Model	All Analyses
Dose	RT dose to constrictor muscles	16	26
	RT dose to superior pharyngeal constrictor (SPC)	16	18
	RT dose to larynx	10	16
	RT dose to middle pharyngeal constrictor (MPC)	10	12
	RT dose to inferior pharyngeal constrictor (IPC)	9	12
	RT dose to esophageal inlet/cricopharnygeus	5	8
	RT dose to oral cavity volume/oral mucosa surface	4	5
	RT dose to parotids	3	7
	RT dose to tongue or base of tongue	3	6
	RT dose to esophagus	2	4
	RT dose to inferior brain stem	1	1
	RT dose to submandibular glands	0	2
Tumor	T-stage	12	20
	Tumor site	10	17
	N-stage	7	11
Patient	Age	12	13
	Smoking history	6	6
	Baseline/acute weight loss	5	8
	Pretreatment or acute dysphagia	3	7
	Body mass index	1	2
	Performance status score	1	4
	Sex	1	3
	Constrictor muscle geometry	1	1
	Alcohol use	0	1
Treatment	Concurrent chemotherapy	9	9
	Surgery-related factors	4	6
	RT fractionation	3	11
	Neck irradiation regimen	2	8
	Chemotherapy drug type	1	3
	Neoadjuvant chemotherapy	1	3
	Adjuvant chemotherapy	1	2
	RT modality	1	2
	Brachytherapy	1	1
	RT field size	0	2
Clinical laboratory tests	Blood or saliva properties	1	1

**Table 6:** Predictive models for OM.

Ref.	Time Frame	Endpoint	Model Features *	Sample Size	Validation Type	Test AUC
[16]	Acute	Increase from RTOG G1-G2	Oral bacteria genetic information	41	Internal	0.646
[17]	Acute	CTCAE G3+ OM	BMI, Combined parotid glands EUD, Oral cavity EUD	132	Internal	0.67
[18]	Acute	CTCAE G3+ OM	Oral cavity D_mean_, Mean RT dose at which 50% of patients experience toxicity (51 Gy), Slope of dose response curve	169	External	0.67
[19]	Acute	CTCAE G3+ OM	Definitive RT, Male, Age, Chemotherapy modality, Chemotherapy drug, Tumor site, Volumes of oral cavity receiving 20-260cGy per fraction in 20cGy/fraction increments	351	Internal	0.71
[20]	Acute	WHO G3+ OM	Age, N-stage, # of cycles of neoadjuvant chemotherapy, V40 (oral cavity)	190	Internal	0.759
[21]	Acute	RTOG G3+ OM	BMI, RLN irradiation, Mucosa surface contour V55	270	Internal	0.782
[22]	Acute	DAHANCA G3+ OM	Extended oral cavity DVH parameters converted into 2 Principal Components, Treatment acceleration	802	Internal	0.808
[23]	Acute	CTCAE G3+ OM	4 cT1-w MR and 1 CECT radiomics texture features extracted from gross tumor volume (primary and nodal tumor)	242	Internal	0.81

* D_mean_ = mean dose, BMI = body mass index, DVH = dose volume histogram, Vx = volume receiving x Gy dose, RLN = retropharyngeal lymph nodes, EUD = equivalent uniform dose, cT1-w MR = contrast T1-weighted magnetic resonance image, CECT = contrast enhanced CT image.

**Table 7:** Predictive models for dysphagia.

Ref.	Time Frame	Endpoint	Model Features *	Sample Size	Validation Type	Test AUC
[93]	Acute	CTCAE G3+ dysphagia	CCT, D2 SPCM, Rs321345_TC(XRCC1) polymorphism	189	Internal	0.6
[94]	Acute	Tube feeding use ≥ 4 weeks	Pre-treatment weight change %, Texture modified diet., ECOG > 0, Tumor site, N-stage ≥ 2, D_mean_ contralateral parotid, D_mean_ oral cavity	334	External	0.624
[95]	Acute	Tube feeding use ≥ 4 weeks	Tumor site, T-stage ≥ 3, Chemotherapy (vs. RT alone), D_mean_ contralateral parotid	225	Internal	0.708
[96]	Acute	Tube feeding use ≥ 4 weeks	BMI, Texture-modified diet, WHO performance scale > 0, Tumor site, T-stage ≥ 2, N-stage ≥ 2, CCT (vs. RT alone), D_mean_ contralateral submandibular gland, D_mean_ contralateral parotid	450	Internal	0.723
[97]	Acute	CTCAE G3+ dysphagia	Definitive RT, Male, Age, IC, No CCT, Chemotherapy drug, Tumor site, Volumes of Pharyngeal mucosa receiving 20-260cGy per fraction in 20cGy/fraction increments	90	External	0.82
[98]	Late	Dysphagia improvement (reduction of at least one grade from CTCAE grade ≥ 3)	D_min_ larynx	90	Internal	0.697
[99]	Late	Aspiration (>25 months)	Age, Neck dissection, D_mean_ MPCM	107	Internal	0.73
[100]	Late	RTOG G2+ dysphagia (6 months)	D_mean_ SPCM, D_mean_ supraglottic larynx	186	External	0.75
[101]	Late	CTCAE G2+ dysphagia (6 months)	D_mean_ oral cavity, D_mean_ SPCM, D_mean_ MPCM, D_mean_ IPCM, Tumor site, Baseline dysphagia score	277	External	0.8
[102]	Late	RTOG G2+ dysphagia (6 months)	D_mean_ SPCM, D_mean_ supraglottic larynx	354	Internal	0.8
[103]	Late	Tube feeding dependence (6 months)	T-stage ≥ 3, N-stage > 0, Baseline weight loss, Accelerated RT, CRT, Neck irradiation	183	External	0.82
[104]	Late	Aspiration or stricture or tube feeding or aspiration pneumonia (>12 months)	Age, V69 Mylo/geniohyoid complex	300	Internal	0.835
[105]	Late	Feeding tube insertion or aspiration (6 months)	Tumor–organ distances for superior, inferior, and medial pharyngeal constrictors, plus mylogeniohyoid, cricopharyngeal muscle, and supraglottic larynx, Clinical feature clusters comprising smoking status, T-stage, N-stage, HPV status, Pathological grade, tumor site, CRT combination, tumor laterality, age, total dose to tumor	200	Internal	0.84
[106]	Late	Tube feeding dependence (6 months)	T-stage ≥ 3, Baseline weight loss > 10%, RT + cetuximab, Accelerated RT, CCT, D_mean_ SPCM, D_mean_ ICPM, D_mean_ contralateral parotid, D_mean_ cricopharyngeal muscle	355	Internal	0.85

* ECOG = Eastern Cooperative Oncology Group performance status, D_mean_ = mean dose, D_x_ = dose to x% of volume, BMI = body mass index, IC = induction chemotherapy, CRT = chemoradiation, CCT = concurrent chemotherapy, [S/M/I]PCM = superior/medial/inferior constrictor muscle, Vx = volume receiving x Gy dose.

## Data Availability

The data presented in this study are available in the Supplementary Materials.

## Supplementary Materials

The following supporting information can be downloaded at https://www.mdpi.com/article/10.3390/cancers15235705/s1, Table S1: Analysis of included OM studies; Table S2: Analysis of included dysphagia studies; Table S3: PRISMA-ScR checklist.

## Appendix A

**Table A1:** Search strategy.

Database	Search String	N Results	Date
Embase	TITLE = (toxicit* OR morbidit* OR “side effect*” OR mucositis OR dysphagia OR deglutition OR swallow* OR “tube feed*” OR ryle* OR enteral OR nasogastric OR intubation OR aspiration OR stricture* OR gastronom* OR “oral intake”) AND (predict* OR model* OR correlat* OR corresp* OR depend* OR assoc* OR relation* OR interact* OR link* OR “risk factors”) TI/AB/KW = (mucositis OR dysphagia OR deglutition OR swallow* OR “tube feed*” OR ryle* OR enteral OR nasogastric OR intubation OR gastronom* OR “oral intake”) AND (radiation OR chemotherap* OR radiotherap* OR chemoradiation OR chemoradiotherap* OR radiochemotherap* OR pharmacotherap* OR “IMRT” OR “VMAT” OR “3DCRT” OR “CRT”) FILTERS Publication Year >= 2000 Abstract OR Article	1093	9/2023
PubMed	(((“Stomatitis”[Mesh] OR “Deglutition Disorders”[Mesh]) AND (“Radiotherapy”[Mesh] OR “Drug Therapy”[Mesh]))) AND ((predict*[Title] OR model*[Title] OR correlat*[Title] OR corresp*[Title] OR depend*[Title] OR assoc*[Title] OR relat*[Title] OR interact*[Title] OR link*[Title] OR “risk*”[Title])) FILTERS Publication Year >= 2000	553	9/2023
Scopus	TITLE = (toxicit* OR morbidit* OR “side effect*” OR mucositis OR dysphagia OR deglutition OR swallow* OR “tube feed*” OR ryle* OR enteral OR nasogastric OR intubation OR aspiration OR stricture* OR gastronom* OR “oral intake”) AND (predict* OR model* OR correlat* OR corresp* OR depend* OR assoc* OR relation* OR interact* OR link* OR “risk factors”) TI/AB/KW = (mucositis OR dysphagia OR deglutition OR swallow* OR “tube feed*” OR ryle* OR enteral OR nasogastric OR intubation OR gastronom* OR “oral intake”) AND (radiation OR chemotherap* OR radiotherap* OR chemoradiation OR chemoradiotherap* OR radiochemotherap* OR pharmacotherap* OR “IMRT” OR “VMAT” OR “3DCRT” OR “CRT”) FILTERS Publication Year >= 2000 Abstract OR Article	701	9/2023
Web of Science	TITLE = (toxicit* OR morbidit* OR “side effect*” OR mucositis OR dysphagia OR deglutition OR swallow* OR “tube feed*” OR ryle* OR enteral OR nasogastric OR intubation OR aspiration OR stricture* OR gastronom* OR “oral intake”) AND (predict* OR model* OR correlat* OR corresp* OR depend* OR assoc* OR relation* OR interact* OR link* OR “risk factors”) TOPIC = (mucositis OR dysphagia OR deglutition OR swallow* OR “tube feed*” OR ryle* OR enteral OR nasogastric OR intubation OR gastronom* OR “oral intake”) AND (radiation OR chemotherap* OR radiotherap* OR chemoradiation OR chemoradiotherap* OR radiochemotherap* OR pharmacotherap* OR “IMRT” OR “VMAT” OR “3DCRT” OR “CRT”) FILTERS Publication Year >= 2000 Abstract OR Article	797	9/2023
